# Analysis and expression of the carotenoid biosynthesis genes from *Deinococcus wulumuqiensis* R12 in engineered *Escherichia coli*

**DOI:** 10.1186/s13568-018-0624-1

**Published:** 2018-06-02

**Authors:** Xian Xu, Liqing Tian, Jiali Xu, Chengjia Xie, Ling Jiang, He Huang

**Affiliations:** 1 School of Pharmaceutical Sciences, Nanjing Tech University, Nanjing, Jiangsu Province China; 2 State Key Laboratory of Materials-Oriented Chemical Engineering, College of Biotechnology and Pharmaceutical Engineering, Nanjing Tech University, Nanjing, Jiangsu Province China; 3 School of Chemical Engineering, Yangzhou Polytechnic Institute, Yangzhou, 225127 Jiangsu Province China; 4 College of Food Science and Light Industry, Nanjing Tech University, Nanjing, Jiangsu Province China

**Keywords:** Lycopene, *Escherichia coli*, Gene regulation, Fermentation optimization

## Abstract

*Deinococcus wulumuqiensis* R12 is a red-pigmented extremophilic microorganism with powerful antioxidant properties that was isolated from radiation-contaminated soil in Xinjiang Uyghur Autonomous Region of China. The key carotenoid biosynthesis genes, *crtE, crtB* and *crtI*, which are related to the cells’ antioxidant defense, were identified in the sequenced genome of R12 and analyzed. In order to improve the carotenoid yield in engineered *Escherichia coli*, the origin of carotenoid biosynthesis genes was discussed, and a strain containing the R12 carotenoid biosynthesis genes was constructed to produce lycopene, an important intermediate in carotenoid metabolism. The gene order and fermentation conditions, including the culture medium, temperature, and light, were optimized to obtain a genetically engineered strain with a high lycopene production capacity. The highest lycopene content was 688 mg L^−1^ in strain IEB, which corresponds to a 2.2-fold improvement over the original recombinant strain EBI.

**Electronic supplementary material:**

The online version of this article (10.1186/s13568-018-0624-1) contains supplementary material, which is available to authorized users.

## Introduction

Lycopene is a representative molecule from the carotenoid family, and is one of the strongest antioxidants known to date. Due to its physiological effects (e.g. immune enhancement, free radical scavenging), lycopene is widely used in various fields, such as medicine, food and cosmetics (Moise et al. 2013; Ciriminna et al. 2016). Lycopene production by microbial fermentation has attracted much attention in recent years because of the identification of biosynthetic genes and the discovery of new highly productive pigment-producing strains. The strains that are used to produce lycopene mainly include microbes that can synthesize lycopene naturally, such as *Blakeslea trispora*, *Erwinia herbicola*, *Rhodotorula* genus, or *Dunaliella salina*, and engineered microbes, such as *Escherichia coli*, *Saccharomyces cerevisiae*, *Candida utilis,* or *Yarrowia lipolytica* (Hernández-Almanza et al. 2016; Mantzouridou and Tsimidou 2008; Miura et al. 1998). A new species with powerful antioxidant capacity, *Deinococcus wulumuqiensis* R12, was screened from an irradiated area in Xinjiang province (Wang et al. 2010). It appears red to the unaided eye because of its production of carotenoids, which is one of the major mechanisms of its radiation resistance. Due to this, the radiation-resistant R12 strain can be used as a new platform for carotenoid synthesis, as well as a model for research on the biological adaptations of extremely radioresistant bacteria.

**Fig. 1:**
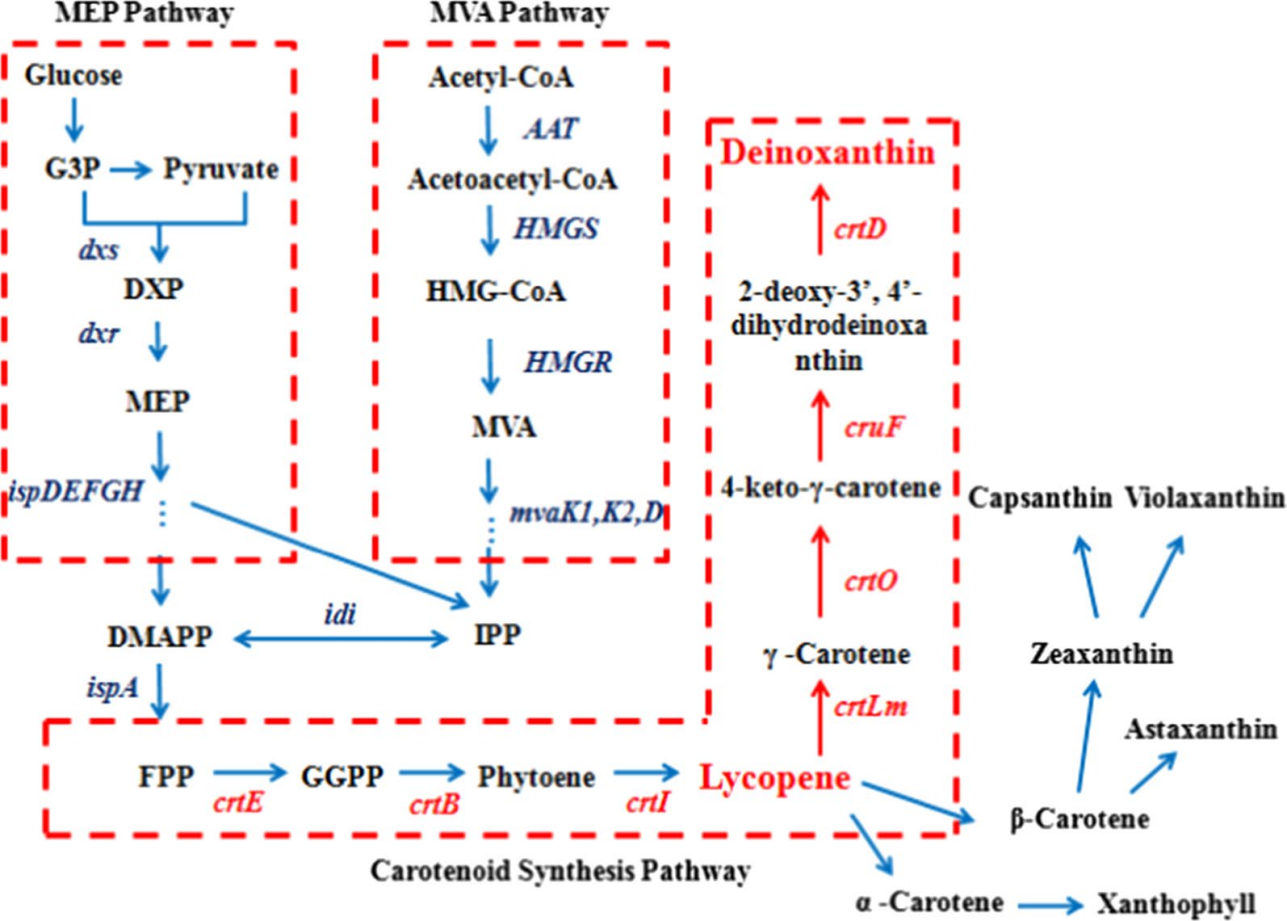
The biosynthesis pathways of lycopene and other carotenoids. The MVA pathway is found in eukaryotic cells, the cytoplasm and mitochondria of plants. The MEP pathway is found in bacteria, other prokaryotes and plastids in plants. The carotenoid synthesis pathway in *Deinococcus radiodurans* R1 was marked with red arrows. *G3P* glyceraldehyde 3-phosphate, *DXP* 1-deoxy-d-xylulose-5-phosphate, *MEP* 2-C-methyl-d-erythritol-4-phosphate, *DMAPP* dimethylallyl diphosphate, *IPP* isopentenyl diphosphate, *HMG-CoA* 3-hydroxy-3-methyl glutaryl coenzyme A, *MVA* mevalonate, *FPP* farnesyl diphosphate, *GGPP* geranylgeranyl diphosphate

There are known two lycopene-synthesis pathways in microorganisms. One is the mevalonate (MVA) pathway, which is present in all known eukaryotic cells and the cytoplasm and mitochondria of plants, and the other is the 2-C-methyl-d-erythritol-4-phosphate (MEP) pathway present in bacteria, other prokaryotes and the plastids of plants (Hernández-Almanza et al. 2016). Lycopene is a typical product of a multi-enzyme catalytic pathway, in which isopentenyl pyrophosphate (IPP), dimethylallyl pyrophosphate (DMAPP) and farnesyl pyrophosphate (FPP) are synthesized by 8 sequential enzymes in the MEP pathway, after which they are converted to lycopene by the three key enzymes geranylgeranyl diphosphate synthase (encoded by *crtE*), phytoene synthase (encoded by *crtB*), and phytoene desaturase (encoded by *crtI*) (Fig. 1). Lycopene can then be converted into a variety of carotenoids and derivatives in different organisms through modification reactions such as cyclizations, oxygenations and dehydrogenations, which makes it one of the most important intermediates in the carotenoid family. With the development of metabolic engineering and synthetic biology, lycopene production by microbial fermentation has gained increasing attention from researchers due to its advantages of lower potential cost and simpler, safer processes. The lycopene biosynthesis genes from various microorganisms, such as *Erwinia uredovora*, *Erwinia herbicola*, *Pantoea ananatis*, *Pantoea agglomerans*, and *Brevibacterium linens*, have been co-expressed in recombinant strains (Yan et al. 2013; Alper et al. 2005; Yoon et al. 2007). Some strategies have improved lycopene production by regulating the expression of key genes, gene knockouts, changing the external conditions, and adding exogenous substances (Yan et al. 2013; Alper et al. 2005; Yoon et al. 2007; Kim et al. 2011; Bhosale 2004; Roukas 2015; Zhu et al. 2015; Matthäus et al. 2014; Arayagaray et al. 2012; Bahieldin et al. 2014). In these genetic engineering strategies, the co-expression of key lycopene synthesis genes in hosts constitutes the traditional approach, which may lead to an imbalance of metabolic fluxes that negatively affects the product yield. It is therefore imperative to preserve the balance of metabolic fluxes in these multi-gene expression systems, which requires intensive study.

In this study, lycopene biosynthesis genes from the newly discovered species *Deinococcus wulumuqiensis* R12 were identified, analyzed, and integrated into a polycistronic plasmid for expression in *Escherichia coli*. Lycopene production of the recombinant strain was investigated in different culture media, and under different temperature and light conditions. Finally, plasmids with the lycopene biosynthesis genes *crtE*, *crtB*, and *crtI* arranged in different order were constructed to study the effect of gene order, which is related to the individual genes’ translation efficiency, on the lycopene yield.

## Materials and methods

### Bacterial strains, plasmids, and growth conditions

All bacterial strains and plasmids used in this study are listed in Table 1. *E. coli* DH5α and *E. coli* BL21 (DE3) cells were used for cloning and gene expression, respectively. *D. wulumuqiensis* R12 (CGMCC 1.8884^T^) (Wang et al. 2010) was grown in TGY medium (10 g L^−1^ of tryptone, 1 g L^−1^ of glucose, and 5 g L^−1^ of yeast extract) at 30 °C. Recombinant *E. coli* cells were grown at 37 °C in Luria–Bertani (LB) medium (10 g L^−1^ of tryptone, 5 g L^−1^ of yeast extract, and 10 g L^−1^ of NaCl), 2×YT medium (16 g L^−1^ of tryptone, 10 g L^−1^ of yeast extract, and 5 g L^−1^ of NaCl), 2× YT + G medium (2× YT medium with 10, 20, 40, 60, 80, or 100 g L^−1^ glycerol), or synthetic medium (SM) [10 g L^−1^ of glycerol, 10 g L^−1^ of glucose, 7.5 g L^−1^ of L-arabinose; 11.2 g L^−1^ of KH_2_PO_4_, 3 g L^−1^ of (NH_4_)_2_HPO_4_, 0.3 g L^−1^ of NaCl, 1 g L^−1^ of MgSO_4_∙7H_2_O, 1.1 g L^−1^ of leucine, 0.7 g L^−1^ of isoleucine, 0.4 g L^−1^ of valine, 1.5 g L^−1^ of threonine, 2 g L^−1^ of lysine, 3.3 g L^−1^ of phenylalanine, 2.2 g L^−1^ of glutamine, and 3.3 g L^−1^ of methionine] (Kim et al. 2011). For lycopene production, a single colony was used to inoculate 50 mL of medium in a 250 mL flask, which was then incubated at 37 °C and 200 rpm for 16 h. Subsequently, 3 mL of the pre-culture was used to inoculate 50 mL of medium and incubated at 37 °C and 200 rpm for 3 h. The cultures were then fermented with or without isopropyl-β-d-thiogalactoside (IPTG, 0–1 mM) under different conditions. Where appropriate, 100 mg L^−1^ of ampicillin was added to promote plasmid retention. Cultivation was conducted in the dark in biological triplicates. To determine the dry cell weight (DCW), 1 mL of the sample was centrifuged (13,000×*g*, 5 min), washed twice with double-distilled water, centrifuged again and dried at 100 °C until constant weight.

**Table 1:** Bacterial strains and plasmids used in this study

Plasmid	Relevant properties	Source
pET-22b	Amp^R^, T7 promoter	Invitrogen
pET-E	Amp^R^, carrying the *crtE* gene from *D. wulumuqiensis* R12	This study
pET-EB	Amp^R^, carrying the *crtE* and *crtB* genes from *D. wulumuqiensis* R12	This study
pET-EBI	Amp^R^, carrying the *crtE*, *crtB* and *crtI* genes from *D. wulumuqiensis* R12	This study
pET-EIB	Amp^R^, carrying the *crtE*, *crtI* and *crtB* genes from *D. wulumuqiensis* R12	This study
pET-BEI	Amp^R^, carrying the *crtB*, *crtE* and *crtI* genes from *D. wulumuqiensis* R12	This study
pET-BIE	Amp^R^, carrying the *crtB*, *crtI* and *crtE* genes from *D. wulumuqiensis* R12	This study
pET-IEB	Amp^R^, carrying the *crtI*, *crtE* and *crtB* genes from *D. wulumuqiensis* R12	This study
pET-IBE	Amp^R^, carrying the *crtI*, *crtB* and *crtE* genes from *D. wulumuqiensis* R12	This study
Strains		
*Deinococcus wulumuqiensis* R12	Aerobic, Gram-positive, non-spore-forming, nonmotile, tetrad-forming coccus; forming reddish-orange, circular, opaque colonies (approx. 1.8–3.8 mm in diameter) after incubation on TGY medium for 14 days at 37 °C	(Wang et al. 2010)
*E. coli* DH5α	deoR endA1 gyrA96 hsdR17 (rK− -mK+) recA1 relA1 supE44 thi-1 Δ (lacZYA-argF) U169 Φ80lacZ ΔM15 F -λ-	Vazyme
*E. coli* BL21(DE3)	F− ompThsdS (rB− mB−) gal dcm (DE3)	Vazyme
EDWe	Amp^R^, *E. coli* BL21(DE3) containing the plasmid pET-22b	This study
EBI	Amp^R^, *E. coli* BL21(DE3) containing the plasmid pET-EBI	This study
EIB	Amp^R^,*E. coli* BL21(DE3) containing the plasmid pET-EIB	This study
BEI	Amp^R^, *E. coli* BL21(DE3) containing the plasmid pET-BEI	This study
BIE	Amp^R^, *E. coli* BL21(DE3) containing the plasmid pET-BIE	This study
IEB	Amp^R^, *E. coli* BL21(DE3) containing the plasmid pET-IEB	This study
IBE	Amp^R^, *E. coli* BL21(DE3) containing the plasmid pET-IBE	This study

### Genome sequencing and bioinformatics analysis of carotenoid-biosynthesis genes from *D. wulumuqiensis* R12

The genomic DNA of *Deinococcus wulumuqiensis* R12 was isolated using a genomic DNA extraction kit (Takara, China). The draft genome sequence of strain R12 was obtained using the Illumina MiSeq platform, which was performed by BGI Tech Solutions Co., Ltd., China, using a paired-end library. This whole-genome shotgun sequence has been deposited with GenBank under the Accession No. APCS00000000 (http://www.ncbi.nlm.nih.gov/nuccore/APCS00000000). The functional annotation of proteins was conducted using different databases, including Gene Ontology (GO, Version:1.419) (Ashburner et al. 2000), Cluster of Orthologous Groups of proteins (COG, Version:20090331) (Tatusov et al. 2003), Kyoto Encyclopedia of Genes and Genomes (KEGG, Version:59) (Kanehisa et al. 2006), and the NR database in GenBank. The secondary metabolite gene clusters were predicted using the antiSMASH (Antibiotics and Secondary Metabolite Analysis Shell) online tool (http://stothard.afns.ualberta.ca/cgview_server/) (Weber et al. 2015). The carotenoid biosynthesis genes from R12 was blasted with the type strain *Deinococcus radiodurans* R1 in GenBank. Multiple sequence alignment was conducted by Vector NTI (Version: 11.5.1). The enzymes in carotenoid biosynthesis encoded by these genes were analyzed by bioinformatics. Theoretical isoelectric point and molecular weight was calculated by Compute pI/Mw tool (http://us.expasy.org/tools/pi_tool.html). SignalP (http://www.cbs.dtu.dk/services/SignalP-1.1/) was used to predict the signal peptide of these enzymes. Transmembrane prediction program TMHMM (http://www.cbs.dtu.dk/services/TMHMM-2.0/) was applied to identify transmembrane regions.

### DNA manipulation and plasmid construction

**Fig. 2:**
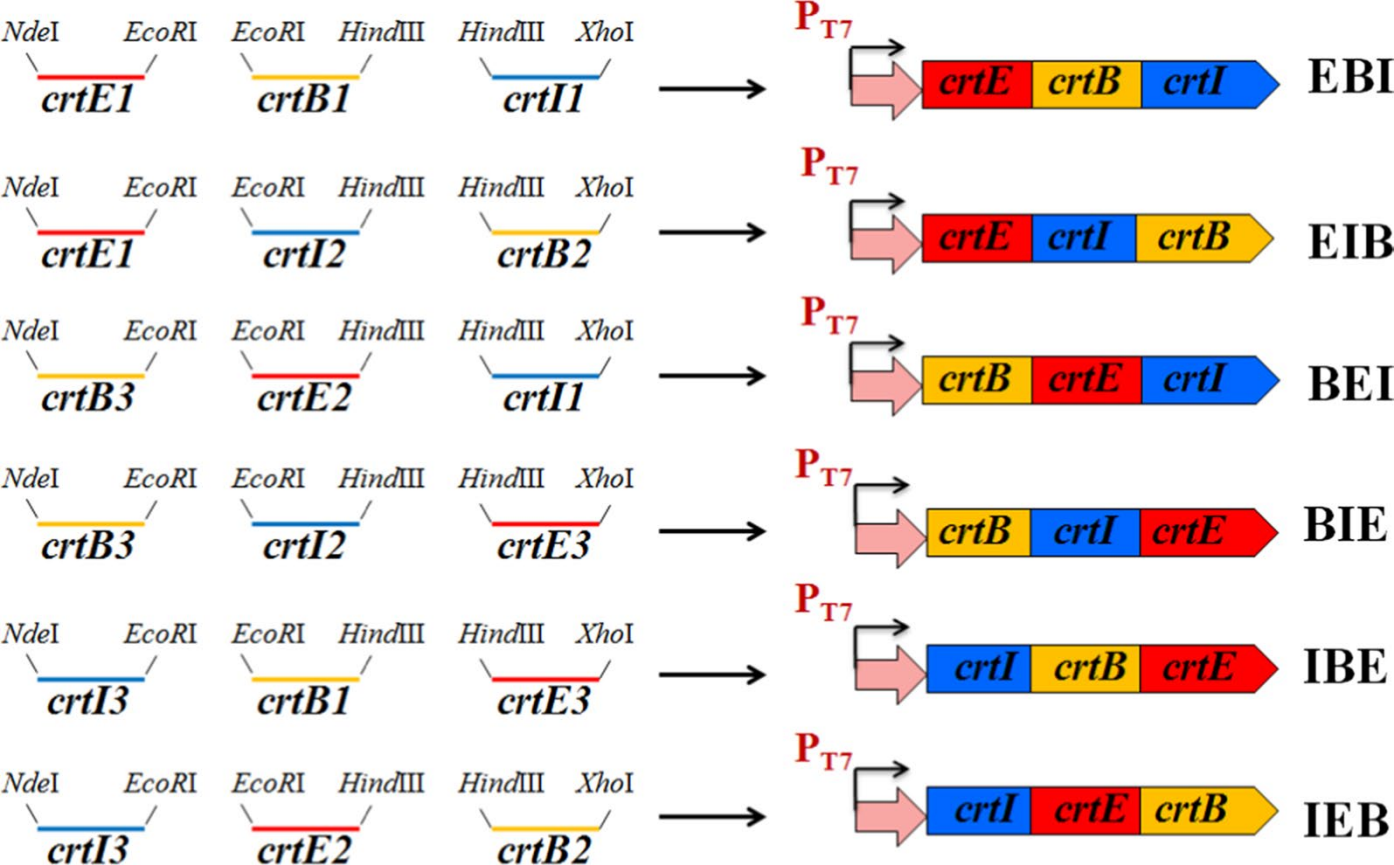
Construction of recombinant plasmids with different gene order of *crtE*, *crtB* and *crtI*. The fragments *crtE1*, *crtE2*, *crtE3*, *crtB1*, *crtB2*, *crtB3*, *crtI1*, *crtI2* and *crtI3* with different restriction enzyme sites were amplified and cloned into pET22b to form six recombinant plasmids with different gene orders of *crtE*, *crtB* and *crtI*. The termination codon TAA of the first and second genes was removed

Fragments encoding *crtE*, *crtB*, and *crtI* were individually amplified from the genomic DNA of *D. wulumuqiensis* R12 using the primers listed in Table 2. The termination codon TAA of *crtB* and *crtI* was removed using appropriately designed primers. The *crtE* PCR fragment was digested with *Nde*I and *EcoR*I, purified, and ligated into the plasmid pET-22b to construct pET-E. The plasmid pET-EB was constructed by digesting the *crtB* fragment with *EcoR*I and *Hin*dIII, purifying, and ligating into plasmid pET-E. The fragment *crtI* was digested with *Hin*dIII and *Xho*I, purified, and ligated into plasmid pET-EB to construct pET-EBI. The fragments *crtE1*, *crtE2*, *crtE3*, *crtB1*, *crtB2*, *crtB3*, *crtI1*, *crtI2* and *crtI3* with different restriction enzyme sites were amplified using the corresponding primers listed in Table 2, and cloned into pET22b to form five recombinant plasmids with a different orders of the three genes, pET-EIB, pET-BEI, pET-BIE, pET-IBE, pET-IEB, in a similar manner as pET-EBI (Fig. 2). Each plasmid was sequenced after each gene ligation, and transferred into *E. coli* BL21 (DE3), resulting in the strains EBI, EIB, BEI, BIE, IBE, and IEB, respectively. pET-22b was introduce into *E. coli* BL21 (DE3) to form EDWe, which was used as the negative control.

**Table 2:** Primers used in this work

Genes	Primer sequence^a^	Restriction enzyme site
*crtE1*	F: 5′- GATCCATATGCGTCCCGAACTG -3′	*Nde*I
	R: 5′- CTTGAATTCCTTCTCCCGCGTCGC -3′	*Eco*RI
*crtB1*	F: 5′- CCGGAATTCGTGACGGAATTTTCGCC -3′	*Eco*RI
	R: 5′- CCCAAGCTTGCCGTGGGCGGCGTC -3′	*Hin*dIII
*crtI1*	F: 5′-CCCAAGCTTATGACATCCCCTCTTCCCTG -3′	*Hin*dIII
	R: 5′- CCGCTCGAGTCAGCGCCGGATGTCG -3′	*Xho*I
*crtI2*	F: 5′- CCGGAATTCATGACATCCCCTCTTCCCTG -3′	*Eco*RI
	R: 5′- CCCAAGCTTGCGCCGGATGTCG -3′	*Hin*dIII
*crtB2*	F: 5′-CCCAAGCTTGTGACGGAATTTTCGCC -3′	*Hin*dIII
	R: 5′- CCGCTCGAGTCAGCCGTGGGCGGCGTC -3′	*Xho*I
*crtB3*	F: 5′- GATCCATATGGTGACGGAATTTTCGCC -3′	*Nde*I
	R: 5′- CTTGAATTCGCCGTGGGCGGCGTC -3′	*Eco*RI
*crtE2*	F: 5′- CCGGAATTCATGCGTCCCGAACTG -3′	*Eco*RI
	R: 5′- CCCAAGCTTCTTCTCCCGCGTCGC -3′	*Hin*dIII
*crtE3*	F: 5′- CCCAAGCTTATGCGTCCCGAACTG -3′	*Hin*dIII
	R: 5′- CCGCTCGAGTCACTTCTCCCGCGTCGC -3′	*Xho*I
*crtI3*	F: 5′- GATCCATATGATGACATCCCCTCTTCCCTG -3′	*Nde*I
	R: 5′- CTTGAATTCGCGCCGGATGTCG -3′	*Eco*RI

^a^Restriction sites are underlined

### Isolation of carotenoids and analytical methods

After cultivation, the cells from 10 mL of culture broth were harvested by centrifugation at 13,000×*g* and 4 °C for 5 min. The resulting cell pellets were collected, washed once with double-distilled water, resuspended in acetone and incubated at 55 °C for 15 min, followed by renewed centrifugation (13,000×*g*, 25 °C, 10 min). The supernatants were used for HPLC analysis. All extraction operations were conducted in the dark.

For HPLC analysis, 20 µL of each supernatant was analyzed using a Venusil XBP C18 column (4.6 × 150 mm, 5 µm; Agela Technologies, USA), kept at 30 °C, and eluted with a mobile phase comprising 80% acetone, 15% methanol, and 5% isopropanol at a flow rate of 1 mL min^−1^ for 40 min. The absorption of the acetone-extracted pigment mixture was detected at 472 nm. Commercial lycopene (Sigma-Aldrich, USA) dissolved in acetone was used as a positive control. All results represent the means ± standard deviations of three independent experiments.

### Nucleotide sequences

The nucleotide sequences of *crtE*, *crtB* and *crtI* from *D. wulumuqiensis* R12 were submitted to the GenBank database with Accession Numbers KP319019, KP319020, and KP319021, respectively.

## Results

### Identification of a carotenoid biosynthetic gene cluster from the genome of *D. wulumuqiensis* R12

*Deinococcus wulumuqiensis* R12 was isolated from radiation-contaminated soils found in Xinjiang Province, China, and the whole genome of R12 was sequenced and analyzed in a previous study (Xu et al. 2013). Functional annotation was completed by blasting predicted genes against the GO, COG and KEGG databases (Additional file 1: Figure S1). During the annotation, we found a number of genes related to metabolic pathways of secondary metabolites and terpenoids. There were 56 genes related to the secondary metabolites biosynthesis, transport and catabolism according to the gene function annotation of the COG database. According to the KEGG database annotation results, 63 genes were found to be related to the metabolism of terpenoids and polyketides (Additional file 1: Figure S1). In addition, the terpenoid pathway, carotenoid biosynthesis pathway, and related genes in the R12 genome were annotated via the KEGG pathway database. Using antiSMASH, 19 secondary metabolic gene clusters were predicted, of which cluster 2 and cluster 13 were associated with the terpene pathway. The similarity of these two gene clusters, which were closest to that of *Deinococcus radiodurans* R1, the type strain of radiation resistant microorganisms, was 31 and 26%, respectively (Additional file 1: Figure S2). These results indicated that the R12 genome indeed contains genes related to the synthesis of terpenes. However, the orientation and distributions of these homologous genes were distinctly different from those of *Deinococcus radiodurans* R1.

There were seven key genes involved in the production of carotenoids in *Deinococcus radiodurans* R1, and the key genes and carotenoid synthesis pathway were marked with red in Fig. 1. The key genes for the synthesis of carotenoids in R12 were identified by BLAST comparison against the genome of R1 (Anderson et al. 1956). The results of bioinformatic analysis of these genes and enzymes were shown in Additional file 1: Table S1. Seven corresponding ORFs in the R12 genome, orf01490, orf00123, orf00124, orf01641, orf02322, orf03006, and orf02323, showed 85.5, 86.3, 86.8, 82.2, 78.7, 81.0 and 90.3% sequence identity to DR1395 (*crtE*, encoding geranylgeranyl diphosphate synthase), DR0862 (*crtB*, encoding phytoene synthase), DR0861 (*crtI*, encoding phytoene desaturase), DR0801 (*crtLm*, encoding lycopene cyclase), DR0091 (*cruF*, encoding carotenoid 1′2′-hydratase), DR2250 (*crtD*, encoding C-3′4′ desaturase) and DR0093 (*crtO*, encoding carotene ketolase) of R1, respectively. Alignment of amino acid sequences showed 85.2, 81.9, 90.9, 81.0, 77.0, 83.7, and 93.9% sequence identity to the corresponding proteins of R1. The isoelectric points of the proteins were between 5 and 10. The C-3′4′ desaturase encoded by orf03006 had a signal peptide, and carotenoid 1′2′-hydratase encoded by orf02322 had seven transmembrane domains. The other corresponding proteins had no signal peptide or transmembrane domains, suggesting that they were intracellular enzymes. The orientation and distribution of the carotenoid biosynthesis genes in the R12 draft genome sequence was illustrated by arrows, compared to those in the whole-genome sequence of R1 (GenBank No. NC001263) (Additional file 1: Figure S3). The carotenoid biosynthesis genes did not constitute a gene cluster in the genomes of these two strains, and were distributed in different loci. Although the genes from R12 were distributed to different scaffolds of the genome, their orientation and order were the same as in the genome of R1.

However, when BLAST analysis of these carotenoids genes was carried out in the NCBI nucleotide database (BLASTN 2.8.0+), there were fewer genes similar to those in the genome of R12. Firstly, there were less than 20 genes similar to the key carotenoid synthesis genes of R12, with a low gene similarity in more than 30% of the cases. In addition, most of these sequences only had sequence-based genomic annotations without experimental verification of gene function. Secondly, the strains with genes similar to those from R12 were grouped in the genera *Deinococcus* and *Thermus*, as well as new genera discovered in recent years. There were obvious differences between these 7 carotenoid biosynthesis genes and similar key genes in other *Deinococcus* species, owing to low identities (36.4–81.6%) and small numbers of similar sequences (Table 3). The protein sequences encoded by these carotenoid biosynthesis genes were also compared between R12 and other *Deinococcus* species (Additional file 1: Table S2). The sequence identities were very low (27.8–88.5%), and some proteins could not be found in some species (especially lycopene cyclase), which was similar to the result of gene alignment. The carotenoid biosynthesis genes and proteins of R12 were obviously different from those of other *Deinococcus* species due to the low sequence identities and low number of available strains for alignment. These carotenoid genes and the corresponding proteins from R12 are therefore worth further study.

**Table 3:** Percentages of sequence identity of carotenoid biosynthesis gene sequences between *D. wulumuqiensis* R12 and other *Deinococcus* spp.

	*crtE* (%)	*crtB* (%)	*crtI* (%)	*crtLm* (%)	*cruF* (%)	*crtD* (%)	*crtO* (%)
*D. radiodurans* R1	85.50	86.30	86.80	82.20	78.70	81.00	90.30
*D. gobiensis* I-0	78.06	67.70	77.90	72.50	68.70	70.40	80.70
*D. actinosclerus* BM2	73.20	66.00	74.00	67.40	56.60	71.00	81.60
*D. swuensis* DY59	75.80	68.90	75.40	62.90	67.00	69.70	81.60
*D. soli* N5	72.90	67.90	74.90	65.30	63.30	72.10	81.00
*D. deserti* VCD115	75.10	59.00	75.00	/	38.10	64.60	75.60
*D. geothermalis* DSM 11300	74.90	65.30	75.00	61.50	61.30	69.40	75.80
*D. puniceus* DY1	74.90	65.70	73.30	60.00	52.50	64.20	80.50
*D. ficus* CC-FR2-10	66.30	62.30	74.50	/	36.80	62.30	75.70
*D. maricopensis* DSM 21211	70.40	62.40	73.10	63.50	59.10	69.30	71.50
*D. proteolyticus* MRP	61.10	55.80	75.00	/	51.80	63.70	74.50
*D. peraridilitoris* DSM 19664	51.20	59.20	68.00	/	36.40	62.20	71.80

### Lycopene production in *E. coli* using carotenoid genes from *D. wulumuqiensis* R12

**Fig. 3:**
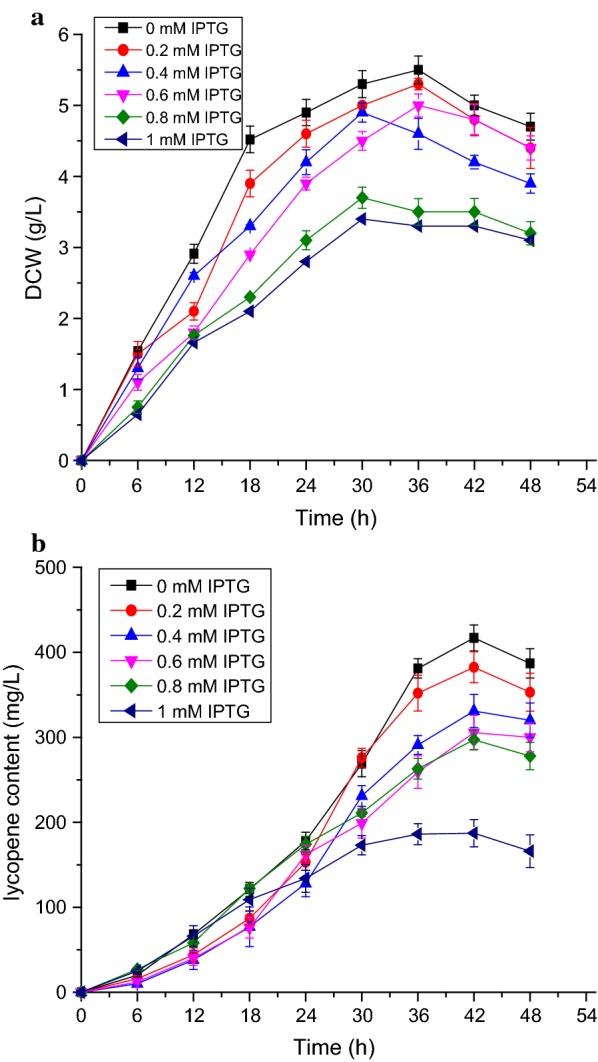
Cell dry weight and lycopene yield of strain EBI induced with different IPTG concentrations. **a** Cell dry weight of EBI in LB medium with the addition of 0–1 mM IPTG at 30 °C for 48 h. **b** Lycopene production of EBI in LB medium with the addition of 0 to 1 mM IPTG at 30 °C for 48 h. The data represent the means of three independent experiments. Error bars represent standard deviations

In carotenoid synthesis, lycopene is formed from FPP by three key enzymes, which are encoded by *crtE, crtB* and *crtI* (Fig. 1). These three genes from *D. wulumuqiensis* R12 were assembled to form pET-EBI, and introduced into *Escherichia coli* BL21 (DE3). Protein expression was induced using IPTG. The acetone supernatants from the EBI strain were separated for 30 min by HPLC, and no lycopene was found in the control strain EDWe carrying the empty vector pET-22b. Colonies of the EBI strain appeared red and the specific peak of lycopene was identified by comparing it with a commercially available authentic lycopene standard. The strain produced a lycopene content of 312 mg L^−1^, proving that *crtE*, *crtB* and *crtI* are indeed the lycopene synthesis genes of *D. wulumuqiensis* R12. The effect of different IPTG concentrations was investigated in the recombinant strain EBI (Fig. 3). The lycopene yield reached the highest value at 42 h, while the biomass reached the maximum at 30–36 h. With the increase of IPTG concentration (0.2 to 1 mM), the biomass and lycopene production both decreased. The highest yield of 418 mg L^−1^ lycopene was achieved at 42 h with no IPTG induction. After 42 h of fermentation, the biomass and lycopene concentration decreased. This decrease may be caused by the consumption of nutrients, the accumulation of harmful metabolites and the pressure on strain growth by the highly hydrophobic lycopene stored in the cell membrane (McNerney and Styczynski 2017). At the same time, cell lysis and the instability of lycopene after long-term fermentation can also lead to a decrease of lycopene yield.

### Optimization of the culture medium for lycopene accumulation

**Fig. 4:**
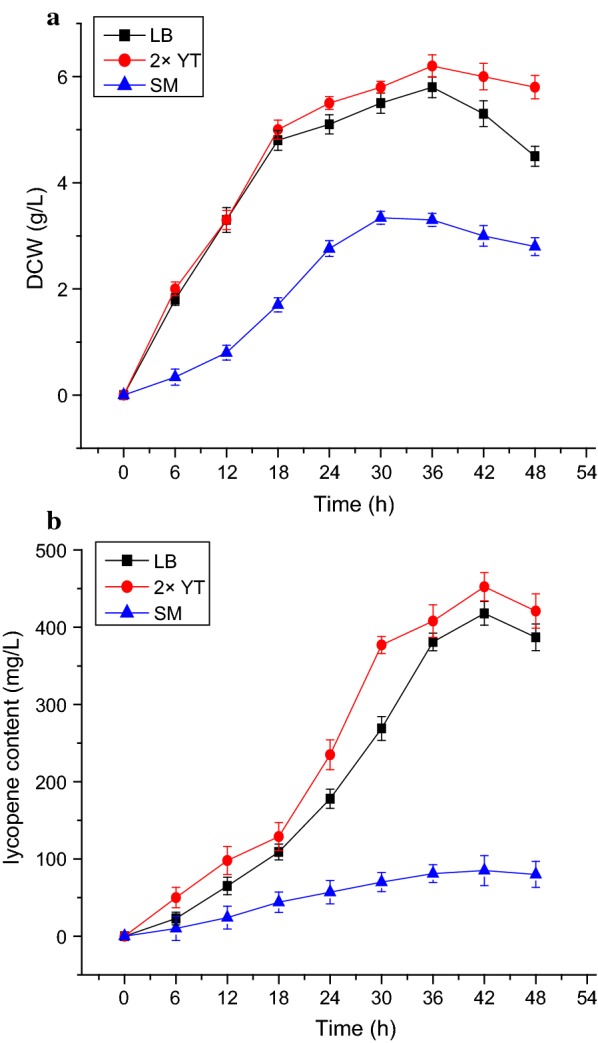
Dry cell weight and lycopene yield of strain EBI in different media. **a** Cell dry weight from strain EBI in LB medium (black squares), 2× YT medium (red circles), and SM medium (blue triangles) with no IPTG, after growth at 30 °C for 48 h. **b** Lycopene content of strain EBI in LB medium (black squares), 2× YT + G medium (red circles), and SM medium (blue triangles) with no IPTG, after growth at 30 °C for 48 h. The data represent the means of three independent experiments. Error bars represent standard deviations

**Fig. 5:**
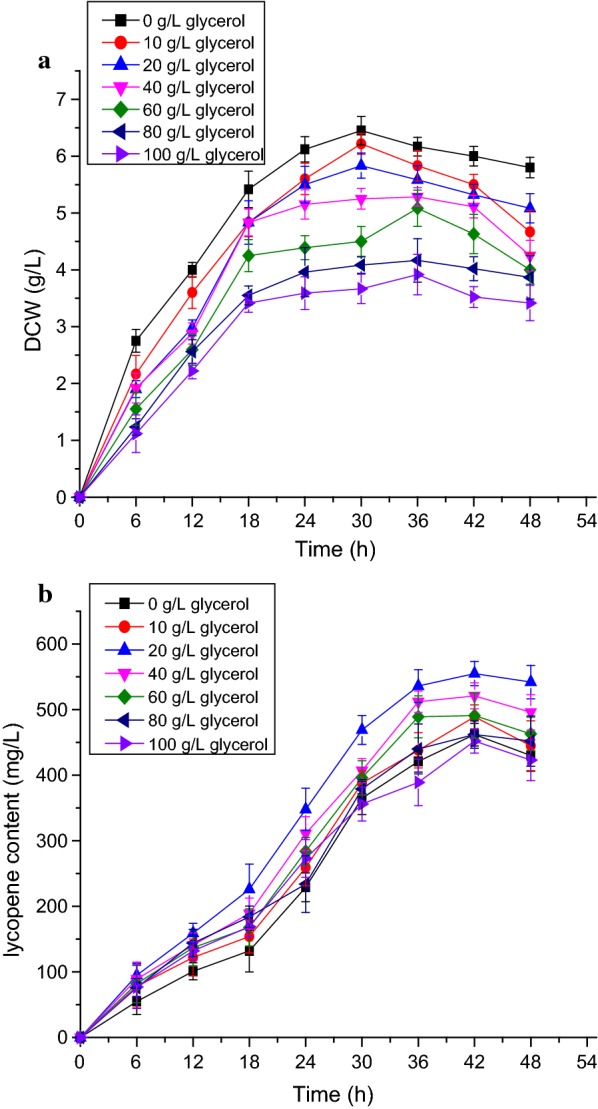
Dry cell weight and lycopene yield of strain EBI with different initial concentrations of glycerol. **a** Cell dry weight of EBI in 2× YT medium with the addition of 0–100 g L^−1^ of glycerol after growth at 30 °C for 48 h. **b** Lycopene production of EBI in 2× YT medium with the addition of 0–100 g L^−1^ of glycerol after growth at 30 °C for 48 h. The data represent the means of three independent experiments. Error bars represent standard deviations

To determine the optimal culture medium, LB, 2× YT, and SM medium were tested. The lycopene production of the EBI strain reached 452.49 mg L^−1^ in 2× YT medium and 418 mg L^−1^ in LB medium. By contrast, the yield in SM medium was only 20 mg L^−1^ (Fig. 4). The effects of additional carbon sources on lycopene production were investigated by adding different concentrations of carbon sources to 2× YT medium (Table 4). The production of lycopene was inhibited by the addition of starch, lactose, and sucrose, while it was increased by the addition of glycerol. Since glycerol had already been proved to increase the yield of lycopene in previous studies (Kim et al. 2011), different concentrations of glycerol (0–100 g L^−1^) were added to 2× YT medium (2× YT + G). As shown in Fig. 5, the biomass reached the maximum of 6.45 g L^−1^ after 30 h, and the lycopene production reached the maximum of 555 mg L^−1^ after 42 h when 20 g L^−1^ glycerol was added. However, the content of lycopene gradually decreased when the initial glycerol concentration was greater than 20 g L^−1^, indicating that the accumulation of lycopene did not require excessive addition of glycerol. Furthermore, cell growth declined rapidly with the increase of initial glycerol concentration, and low levels of biomass limited the lycopene production. These results demonstrated that among the culture media tested in this work, the 2× YT + G medium (20 g L^−1^) was most suitable for the production of lycopene.

**Table 4:** Effects of different auxiliary carbon sources (10 g/L in 2× YT medium) on the lycopene yield of the strain EBI

Auxiliary carbon source	Glucose	Glycerol	Fructose	Starch	Lactose	Sucrose
Lycopene content (mg L^−1^)	371 ± 9.1	481 ± 8.9	449 ± 12.3	295 ± 3.6	183 ± 7.7	214 ± 10.9
DCW	6.2 ± 0.28	5.8 ± 0.12	5 ± 0.3	4.5 ± 0.28	3.3 ± 0.11	4.1 ± 0.17

### The effects of temperature on cell growth and lycopene production

**Fig. 6:**
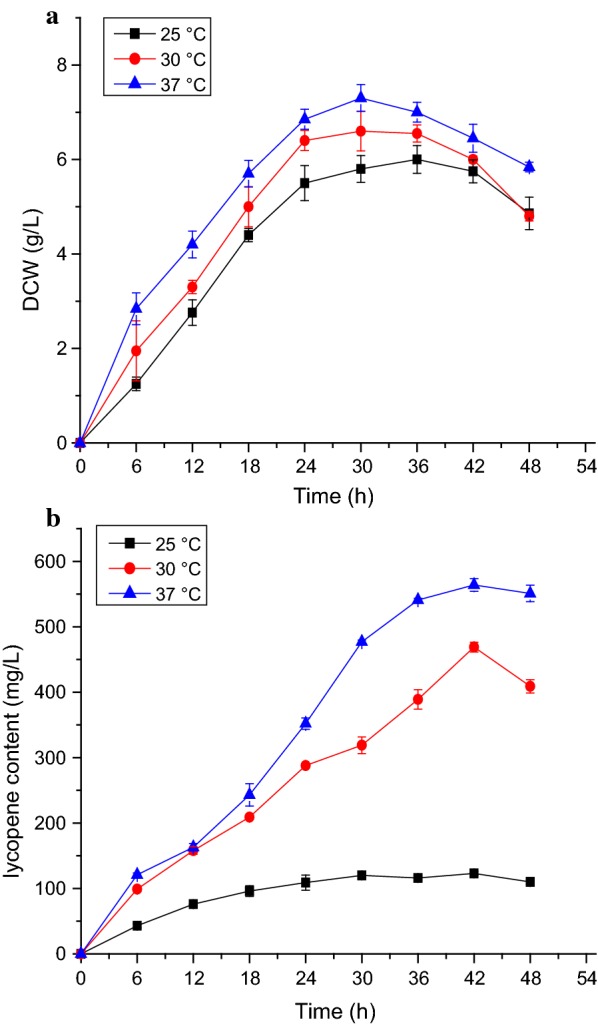
Dry cell weight and lycopene yield of strain EBI at different temperatures. **a** Cell dry weight of EBI in 2× YT + G medium (20 g L^−1^ glycerol) after growth at 25, 30, and 37 °C for 48 h. **b** Lycopene production of EBI in 2× YT + G medium (20 g L^−1^ glycerol) after growth at 25, 30, and 37 °C for 48 h. The data represent the means of three independent experiments. Error bars represent standard deviations

Temperature is considered the main physical element that directly influences the bacterial growth rate and thus plays an important role in the biosynthesis of carotenoids. Three temperatures (25, 30, and 37 °C) were assessed according to previous studies (Kim et al. 2009). As shown in Fig. 6, 37 °C was the best temperature for the growth of the EBI strain according to the DCW results. The highest DCW was 7.3 g L^−1^ at 37 °C after cultivation for 30 h. Moreover, the total lycopene content was much higher at 37 °C than at 30 or 25 °C. The highest lycopene content was 564 mg L^−1^ at 37 °C after cultivation for 42 h. The DCW and lycopene content were the lowest at 25 °C, and the lycopene yield was also especially markedly lower at this temperature. The high biomass obtained at 37 °C may explain the high lycopene content in the cultures. The lycopene content decreased after 42 h of cultivation, suggesting that cultivation at 37 °C for 42 h is optimal for biomass accumulation and lycopene production.

### The effect of light on cell growth and lycopene production

**Fig. 7:**
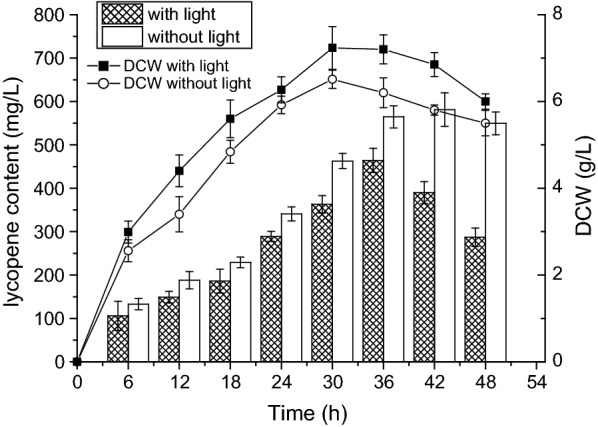
Dry cell weight and lycopene yield of strain EBI with or without light. The shake flasks were exposed to LEDs (filled squares), or were incubated and without light (open circles) at 37 °C for 48 h

Light affects many biological activities such as microbial growth, morphogenesis, and biosynthesis of reduced hydrogen equivalents in living organisms (Chen and Chang 2006; Bohne and Linden 2002). In addition, lycopene is a light-sensitive product. Therefore, the influence of light on lycopene biosynthesis was evaluated. The shake flasks were wrapped in silver paper to protect lycopene in our system. As shown in Fig. 7, the shake flasks were exposed to 40 W of LEDs to assess the influence of light. The strain produced the highest lycopene content (581.2 mg L^−1^) after 42 h of fermentation in the dark, while the biomass was higher under the influence of light. The maximum biomass reached 7.23 g L^−1^ under LED lights at 30 h. These results indicate that light has a non-negligible effect on lycopene accumulation.

### Optimal conditions for lycopene production in shake flasks

**Fig. 8:**
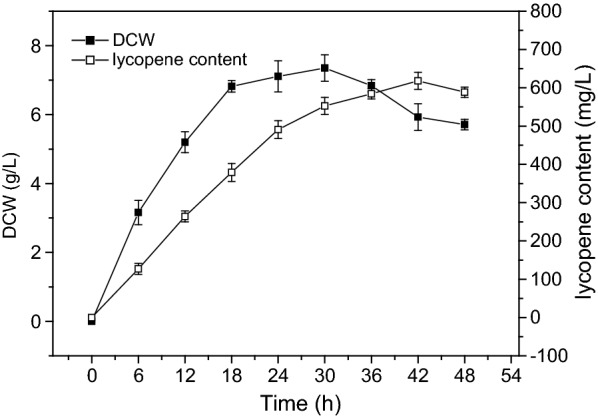
Dry cell weight and lycopene yield of strain EBI under optimized conditions. Dry cell weight (filled squares) and lycopene content (open squares) of the EBI strain under the optimal conditions in shake flask culture. The values are the averages ± standard deviations from three independent experiments

Based on the results of fermentation optimization, the temperature was fixed at 37 °C during the entire cultivation process, and 2× YT medium was used for seed cultivation for 12 h. The preculture was then used to inoculate 50 mL of fresh 2× YT + G medium (with 20 g L^−1^ glycerol) in 250-mL shake flasks. Cultivation was conducted in the dark. As shown in Fig. 8, the biomass increased quickly during the first 18 h of cultivation, then increased slowly, and reached a maximum of 7.35 g L^−1^ at 30 h. The lycopene content increased at the beginning, reaching a maximum at 42 h (618 mg L^−1^), and then gradually decreased. Compared with the original conditions, the biomass of EBI increased 1.99 times and the yield of lycopene improved 1.98-fold after optimization.

### Construction of recombinant plasmids with different *crt* gene order

The DNA fragments encoding *crtE, crtB* and *crtI* were amplified and assembled to from the plasmids pET-EIB, pET-BEI, pET-BIE, pET-IBE, and pET-IEB (Fig. 2), which were transferred into *E. coli* BL21(DE3), resulting in the recombinant strains EIB, BEI, BIE, IBE, and IEB, respectively. Acetone extracts from these strains were analyzed for lycopene content by HPLC (Table 5). The strain BEI had the lowest lycopene content of 228 mg L^−1^. By contrast, the lycopene production of the IEB strain reached up to 688 mg L^−1^, which was the highest of all six strains and more than three times higher than that of the lowest strain.

**Table 5:** Lycopene production of the six recombinant strains with different *crt* gene order

Strain	EBI	EIB	BEI	BIE	IEB	IBE
Lycopene content (mg L^−1^)	605 ± 12	583 ± 15	228 ± 9	373 ± 16	688 ± 10	529 ± 18

The strains are named according to the gene order of E: *crtE,* B: *crtB*, and I: *crtI*

## Discussion

**Fig. 9:**
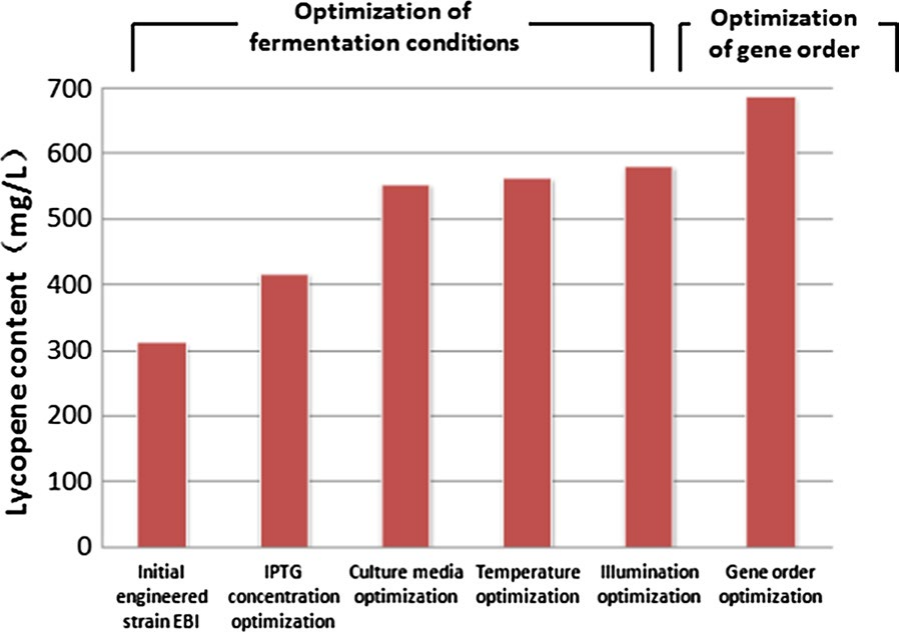
Lycopene production was improved by the combined optimization of culture conditions and gene order. The *E. coli* strain EBI produced a high content of lycopene after twin optimization of fermentation conditions and gene expressing levels

Many efforts have been made to improve the yield of lycopene by engineering bacteria, mostly via the expression of exogenous *crtE*, *crtB* and *crtI* genes for lycopene synthesis from *Erwinia* to *Pantoea* species. Yoon et al. constructed engineered *E. coli* strains harboring lycopene genes from *Pantoea agglomerans* and *Pantoea ananatis*, which produced 60 and 35 mg L^−1^ of lycopene, respectively (Yoon et al. 2007). When the genes *crtE*, *crtB* and *crtI* from *Erwinia uredovora* were integrated into *Candida utilis*, it produced a lycopene yield of 758 μg g^−1^ DCW (Miura et al. 1998). Matthaus et al. constructed a plasmid harboring *crtB* and *crtI* from *Pantoea ananatis* and transformed *Yarrowia lipolytica*, which produced 16 mg g^−1^ DCW of lycopene (Matthäus et al. 2014). When the lycopene synthesis genes from different bacteria were cloned into the pGAPZB plasmid and introduced into *Pichia pastoris* X33, the recombinant strain showed a lycopene production of 73.9 mg L^−1^ (Bhataya et al. 2009). Bahieldin et al. constructed a plasmid harboring the *crt* genes from *Pantoea ananatis* under the control of the ADH2 promoter and introduced it into *Saccharomyces cerevisiae*, which produced a yield of 3.3 mg lycopene g^−1^ DCW (Bahieldin et al. 2014). Thus, diverse sources of lycopene synthesis genes expressed in different hosts resulted in different lycopene yields. However, the lycopene synthesis genes from extremophilic radiation-resistant microorganisms were rarely investigated. In this work, the lycopene synthesis genes from the recently isolated extremophilic microorganism *Deinococcus wulumuqiensis* R12 were analyzed and cloned in *E. coli*. The transgenic *E. coli* strain EBI produced a high content of lycopene after twin optimization of fermentation conditions and gene expressing levels (Fig. 9), and thus provides a new microbial gene source for lycopene synthesis and lays a good foundation for improving lycopene production in engineered *Escherichia coli*.

In prokaryotic expression systems, the strong inducer IPTG exacerbates the toxicity of haloalkane substrates, causing damage to the *E. coli* host, which often bears a metabolic burden due to the recombinant plasmid it contains. Excess IPTG can result in non-trivial economic losses and toxic effects, including reduced cell growth and lower recombinant protein concentration (Papaneophytou and Kontopidis 2014). In our study, when IPTG was not added at all, the lycopene content and cell growth were close to the highest. With the increase of IPTG concentration, the lycopene content and cell growth gradually decreased. Under high levels of protein production, the *E. coli* cells bear a negative pressure known as the metabolic burden or metabolic load, which is attributed to the overconsumption of metabolic precursors (e.g., amino acids, adenosine triphosphate, FPP) to form non-essential foreign proteins, as well as the maintenance and replication of recombinant plasmid vectors (Dvorak et al. 2015; Mairhofer et al. 2013). Low IPTG concentrations can result in efficient induction, and leaky expression sometimes occurs even when IPTG is not added, which allows for sufficient expression of genes within the pathway to achieve a good yield. Similar inducer concentrations that allow full gene expression have been reported (Kim et al. 2011; Bahieldin et al. 2014; Kim et al. 2009; Zhang et al. 2015b). In some cases, tuning the IPTG concentration by reducing it dramatically or even not adding any inducer can improve the host’s fitness, although the mechanism driving the induction of T7 RNAP expression in the absence of IPTG is not clear. Here, we showed that culturing *E. coli* cells in LB medium in the absence of the inducer IPTG could provide a cost-effective, simple and competitive alternative for the production of lycopene.

Optimization of the culture medium is a useful method to enhance lycopene production. In this study, the use of glycerol as an auxiliary carbon source greatly improved lycopene production, which may be due to a higher acetate concentration in the cultures grown on glucose than in the ones grown on glycerol. At high concentrations, acetate acts as an inhibitory metabolite, lowering carotenoid production. Moreover, glucose has been reported to catabolically repress the T7 promoter in the recombinant system we used for lycopene synthesis (Yang and Guo 2014; Guzman et al. 1995).

Temperature is one of the most important environmental factors affecting the growth and development of *E. coli*. In protein expression systems based on *E. coli*, temperature affects both induction and protein expression. Although it was found that lower temperatures favor more lycopene formation (Kim et al. 2009; Vadali et al. 2005; Lee et al. 2004), when the strain EBI was grown at 37 °C the lycopene content and DCW were both higher than at either 30 or 25 °C. Low temperatures decrease the rate of nutrient consumption, and thus some metabolic processes, such as protein synthesis, slow down. Conversely, appropriately high temperatures can promote cell growth, balance enzyme expression and increase the activities of enzymes. It is well-known that carotenoids are important for the protection against photo-oxidative damage in non-photosynthetic organisms. Many non-phototrophic bacteria and fungi rely on carotenoids for protection when growing exposed to light and air (Marova et al. 2012). As with other carotenoids, the stability of lycopene is affected by light. Under illumination, lycopene decomposes via isomerization and oxidation, which protects the cells from oxidative damage caused by exposure to strong light, but also decreases the concentration of lycopene in the cells (Hernández-Almanza et al. 2016).

The efficiency of multi-gene expression systems is mainly affected by promoters, transcription factors, and translation levels. Nevertheless, the gene order is also important. Within an operon, the transcription efficiency of a gene decreases as its position moves away from the promoter. The expression of a gene at the first position is therefore higher than that of an identical gene at the second position, which should be higher than that of an identical gene at the third position, and so on (Han et al. 2011). A novel approach for metabolic pathway optimization, oligo-linker mediated assembly (OLMA), was applied in the lycopene synthetic pathway to swap the order of *crtE*, *crtB* and *crtI*, which led to selection of the best strain EBI, the lycopene yield of which was 36 times higher than that of the least productive strain IEB (Zhang et al. 2015a). In our study, the productivity of strain IEB was 3 times higher than that of the least productive strain BEI, which suggested that the order of genes had a great influence on lycopene synthesis. An improper gene order can result in a severe imbalance in the pathway, which in turn affects the product yield. Through sequential control of the downstream, upstream, and competing pathways of farnesyl diphosphate (FPP) via a predetermined order of key genes in the crucial metabolic node in the biosynthesis of terpenoids, a carotenoid production of 1156 mg L^−1^ (20.79 mg g^−1^ DCW) was achieved (Xie et al. 2015). These strategies indicate that multi-gene expression requires the orderly arrangement of genes to balance their translation levels. Combined with the size and expression of enzymes, a high level of synergy is needed to achieve higher yields.

Phytoene desaturase (PDS, encoded by *crtI*), the first enzyme involved in phytoene conversion to colored carotenoids, catalyzes a rate-limiting step in carotenoid biosynthesis (Chamovitz et al. 1993). The catalytic functions of bacterial phytoene desaturases are diverse, which can lead to low lycopene concentrations because of its poor catalytic specificity. Stickforth et al. demonstrated that high phytoene desaturase concentrations or a low phytoene supply favor the formation of lycopene (Stickforth and Sandmann 2007). Ostrov et al. introduced the lycopene production pathway into a modular biosensor and found that after adding two copies of lycopene synthase (encoding by *crtI*), lycopene production increased more than three times (Ostrov et al. 2017). Among the six strains in our study, the lycopene yield of IEB was the highest. This is probably due to the fact that the *crtI* gene was closest to the promoter, which increased its translation efficiency and the final substrate conversion rate to lycopene. At the same time, lycopene is only synthesized from FPP after successful multi-gene expression of *crtE*, *crtB* and *crtI*, which means that balanced gene expression is needed to avoid excessive accumulation of intermediate products that can inhibit cell growth.

In conclusion, a recombinant strain with a new source of lycopene synthesis genes from the radiation resistant microorganism *Deinococcus wulumuqiensis* R12 was constructed. We found some important differences between these lycopene synthesis genes and other homologous microbial genes, which merits further study. After optimization of culture media, temperature and illumination, the lycopene content of strain EBI reached 618 mg L^−1^ in 2× YT + G medium (with 20 g L^−1^ glycerol), after 42 h of fermentation in the dark at 37 °C. Finally, six recombinant strains with different *crt* gene orders were constructed, and the highest lycopene content was 688 mg L^−1^ in strain IEB, which was about three times higher than that of the lowest strain BEI, underscoring the effect of gene regulation on lycopene synthesis. Taken together, the strain IEB was improved 2.2-fold compared to the original recombinant strain EBI. Our results will provide new guidance for the synthesis, regulation and industrial production of lycopene and other carotenoids.

## Additional file

**Additional file 1: Figure S1.** Gene function annotations of R12 genome in GO, COG and KEGG databases. **Figure S2.** The antiSMASH analysis results of R12 genome. **Figure S3.** The orientation and distribution of the carotenoid biosynthetic genes from the R1 and R12. **Table S1.** Bioinformatic analysis of key enzymes of carotenoid biosynthesis in R12. **Table S2.** Percentages of sequence identity of proteins in carotenoid biosynthesis between *D. wulumuqiensis* R12 and other *Deinococcus spp*.

## Abbreviations

MVA: mevalonate
MEP: 2-C-methyl-d-erythritol-4-phosphate
IPP: isopentenyl diphosphate
DMAPP: dimethylallyl diphosphate
FPP: farnesyl diphosphate
G3P: glyceraldehyde 3-phosphate
DXP: 1-deoxy-d-xylulose-5-phosphate
HMG-CoA: 3-hydroxy-3-methyl glutaryl coenzyme A
GGPP: geranylgeranyl diphosphate
IPTG: isopropyl-β-d-thiogalactoside
DCW: dry cell weight
GO: gene ontology
COG: Cluster of Orthologous Groups of proteins
KEGG: Kyoto Encyclopedia of Genes and Genomes
antiSMASH: Antibiotics and Secondary Metabolite Analysis Shell
OLMA: oligo-linker mediated assembly
