# Supplementary material for: Analysis and expression of the carotenoid biosynthesis genes from Deinococcus wulumuqiensis R12 in engineered Escherichia coli
Source: AMB Express. 2018 Jun 2;8:94. doi: 10.1186/s13568-018-0624-1 (PMC5984946; doi:10.1186/s13568-018-0624-1)
Supplement: Supplementary file 1 — Additional file 1: Figure S1. Gene function annotations of R12 genome in GO, COG and KEGG databases. Figure S2. The antiSMASH analysis results of R12 genome. Figure S3. The orientation and distribution of the carotenoid biosynthetic genes from the R1 and R12. Table S1. Bioinformatic analysis of key enzymes of carotenoid biosynthesis in R12. Table S2. Percentages of sequence identity of proteins in carotenoid biosynthesis between D. wulumuqiensis R12 and other Deinococcus spp. [file 13568_2018_624_MOESM1_ESM.docx]

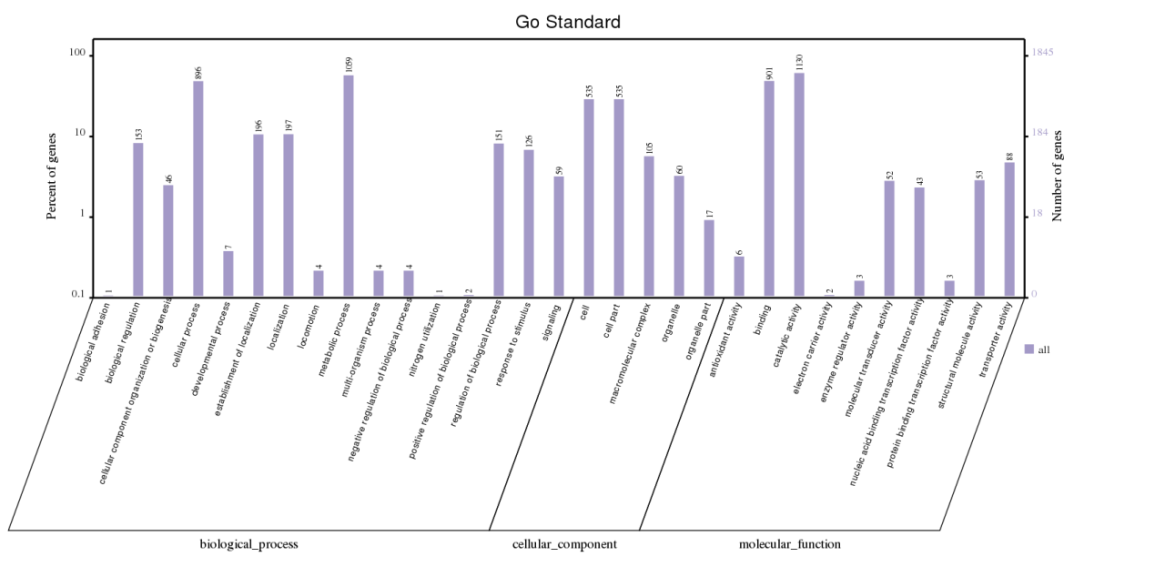


**a**


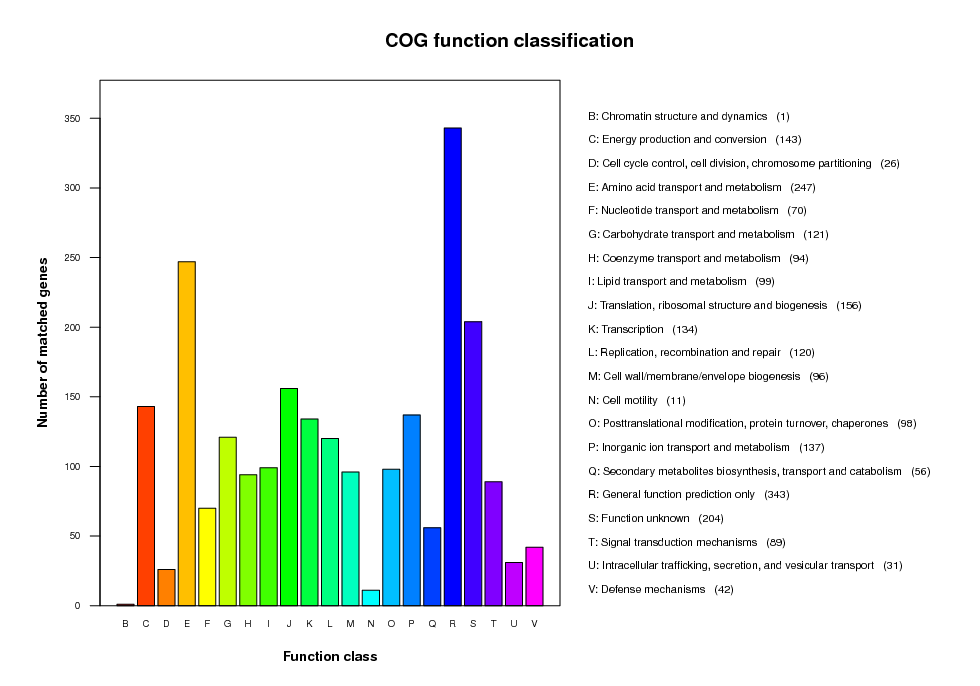


**b**


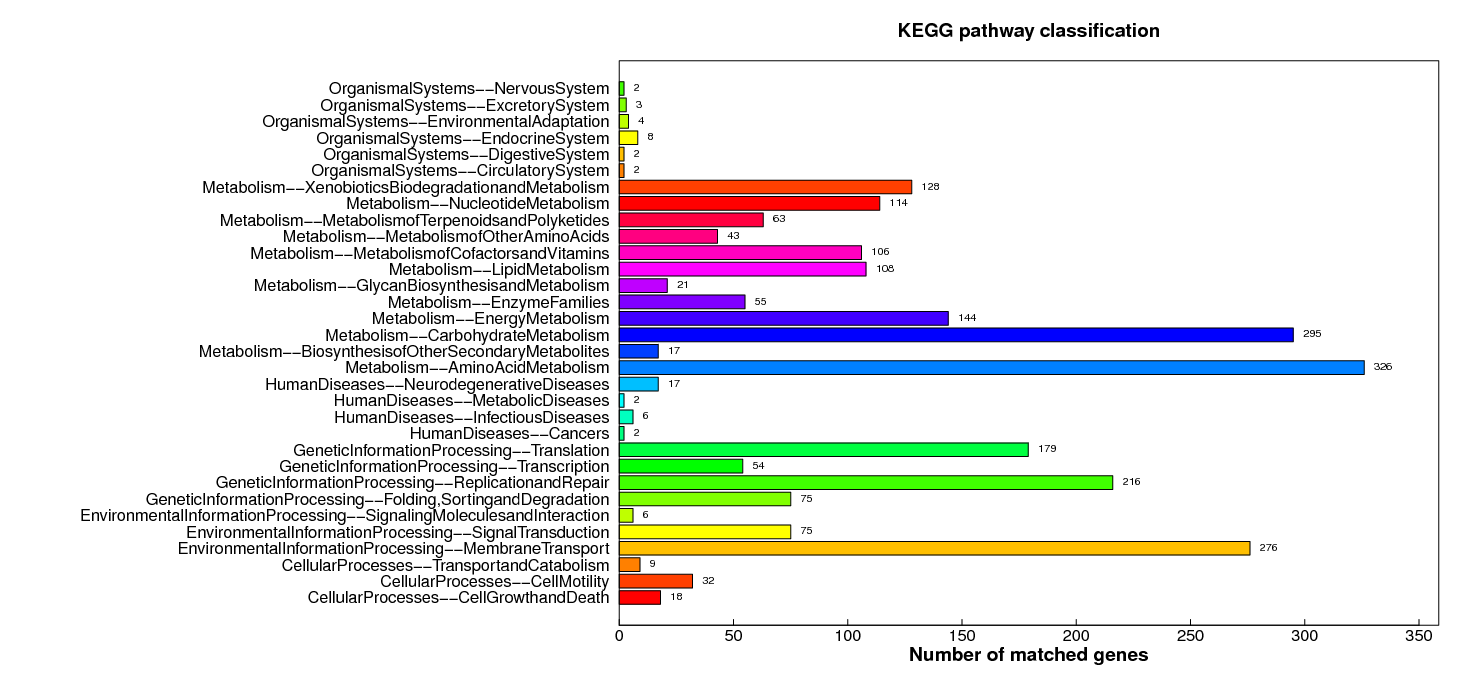


**c**

**Figure S1** Gene function annotations of R12 genome in GO, COG and KEGG databases

a.GO annotation of R12 genome; b. COG annotation of R12 genome; c. KEGG annotation of R12 genome

**a**


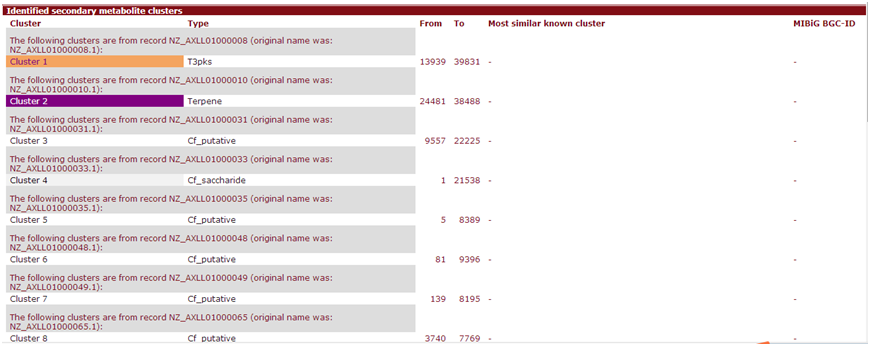

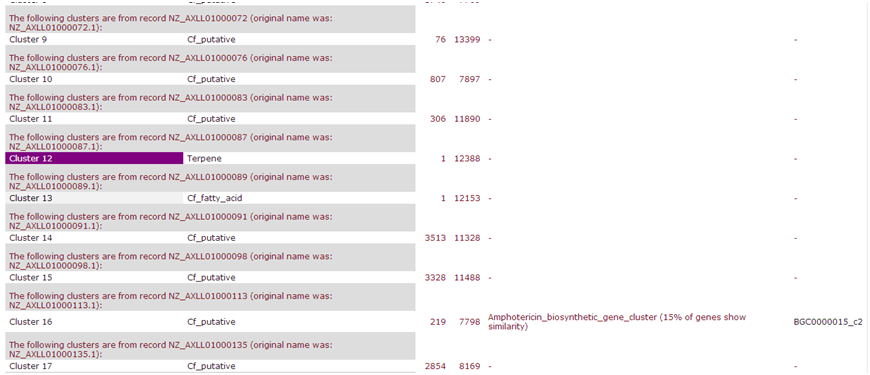

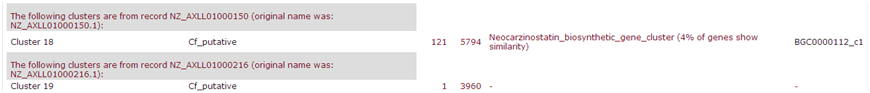


**b**


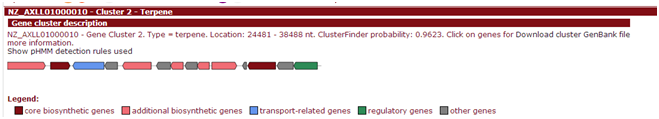

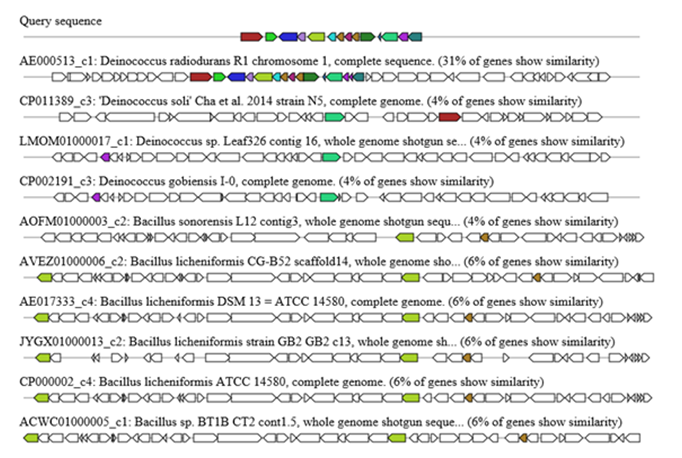


**c**


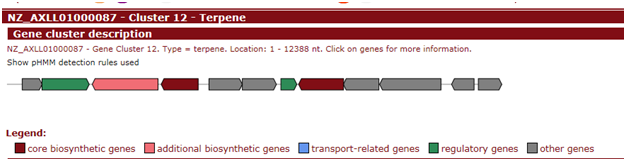

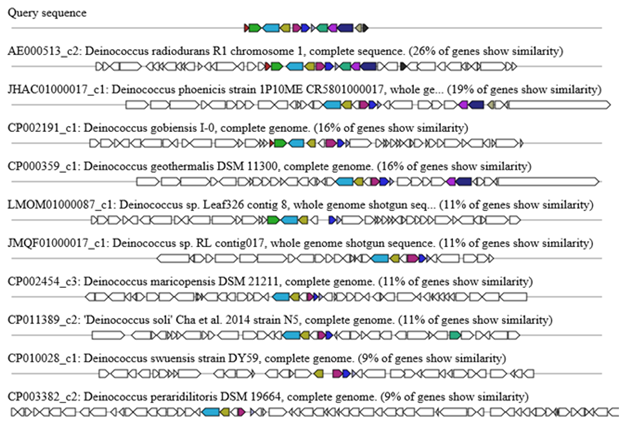


**Figure S2** The antiSMASH analysis results of R12 genome

a. Overall prediction results of R12 genome by antiSMASH; b. Prediction results of gene cluster 2 in R12 genome by antiSMASH; c. Prediction results of cluster1 gene cluster 12 in R12 genome by antiSMASH


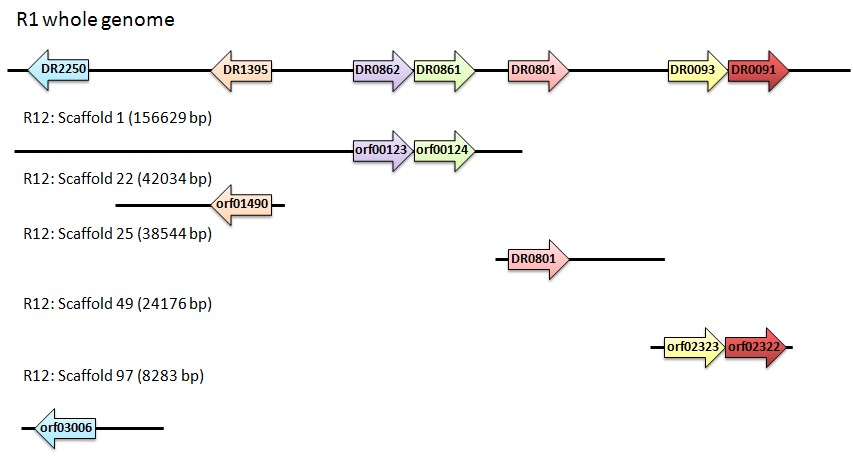


**Figure S3** The orientation and distribution of the carotenoid biosynthetic genes from the R1 and R12

**Table S1** Bioinformatic analysis of key enzymes of carotenoid biosynthesis in R12

| ORFs | Gene | Enzyme | Length of amino acid sequence | PI | KD | Signal peptide | Transmembrane domain | Nucleotides identities (%)^a^ | Amino acid identities (%)^b^ |
| --- | --- | --- | --- | --- | --- | --- | --- | --- | --- |
| orf01490 | *crtE* | geranylgeranyl diphosphate synthase | 329 | 5.52 | 36137.20 | N | N | 85.5 % | 85.2 % |
| orf00123 | *crtB* | phytoene synthase | 308 | 6.33 | 33983.49 | N | N | 86.3 % | 81.9 % |
| orf00124 | *crtI* | phytoene dehydrogenase | 548 | 6.36 | 60335.78 | N | N | 86.8 % | 90.9 % |
| orf01641 | *crtLm* | lycopene cyclase | 405 | 10.34 | 42994.08 | N | N | 82.2 % | 81.0 % |
| orf02322 | *cruF* | carotenoid 1’2’-hydratase | 312 | 10.44 | 33200.43 | N | 7 | 78.7 % | 77.0 % |
| orf03006 | *crtD* | C-3’4’ desaturase | 467 | 10.10 | 49990.03 | Y | N | 81.0 % | 83.7 % |
| orf02323 | *crtO* | carotene ketolase | 511 | 6.54 | 56643.38 | N | N | 90.3 % | 93.9 % |

^a^ Identity is defined as percentage of nucleotides that are identical between *D. wulumiqiensis* R12 and *D. radiodurans* R1.

^b^ Identity is defined as percentage of amino acid that are identical between *D. wulumiqiensis* R12 and *D. radiodurans* R1.

**Table S2** Percentages of sequence identity of proteins in carotenoid biosynthesis between *D. wulumuqiensis* R12 and other *Deinococcus* spp.

|  | Protein encoded by orf01490 | Protein encoded by orf00123 | Protein encoded by orf00124 | Protein encoded by orf01641 | Protein encoded by orf02322 | Protein encoded by orf03006 | Protein encoded by orf02323 |
| --- | --- | --- | --- | --- | --- | --- | --- |
| *D. radiodurans* R1 | 85.2 % | 81.9 % | 90.9 % | 81.0% | 77.0% | 83.7 % | 93.9 % |
| *D. gobiensis* I-0 | / | 46.4 % | 71.7 % | 63.1 % | 50.0 % | 71.2 % | 85.7 % |
| *D. actinosclerus* BM2 | 72.5 % | 64.6 % | 75.6 % | / | 60.5 % | 72.9 % | 88.5 % |
| *D. swuensis* DY59 | 72.6 % | 51.9 % | 75.5 % | 53.7 % | 63.6 % | 73.0 % | 86.7 % |
| *D. soli* N5 | 72.2 % | 64.6 % | 76.1 % | 49.9 % | 59.6 % | 73.0 % | 88.1 % |
| *D. deserti* VCD115 | 75.1 % | 56.5 % | 78.4 % | / | 31.7 % | 67.2 % | 81.8 % |
| *D. geothermalis* DSM 11300 | 75.1 % | 63.3 % | 78.5 % | 57.4 % | 60.6 % | 66.4 % | 82.5 % |
| *D. puniceus* DY1 | 76.3 % | 61.3 % | 74.6 % | 46.6 % | 62.0 % | 66.1 % | 84.4 % |
| *D. ficus* CC-FR2-10 | 68.4 % | 51.1 % | 76.5 % | / | 28.1 % | 64.5 % | 77.5 % |
| *D. maricopensis* DSM 21211 | 66.9 % | 58.4 % | 72.5 % | 57.6 % | 48.6 % | 65.3 % | 76.6 % |
| *D. proteolyticus* MRP | 66.8 % | 33.0 % | 74.8 % | / | 32.2 % | 65.4 % | 76.2 % |
| *D. peraridilitoris* DSM 19664 | 58.1 % | 55.8 % | 68.5 % | / | 27.8 % | 63.1 % | 71.5 % |
